# Wastewater irrigation and *Trichoderma* colonization in tomato plants: effects on plant traits, antioxidant activity, and performance of the insect pest *Macrosiphum euphorbiae*

**DOI:** 10.1007/s11356-024-32407-w

**Published:** 2024-02-14

**Authors:** Vincenzo Trotta, Daniela Russo, Anna Rita Rivelli, Donatella Battaglia, Sabino Aurelio Bufo, Vittoria Caccavo, Pierluigi Forlano, Filomena Lelario, Luigi Milella, Lorenzo Montinaro, Laura Scrano, Monica Brienza

**Affiliations:** 1 Scuola di Scienze Agrarie, Forestali, Alimentari e Ambientali, Università della Basilicata, via dell’Ateneo Lucano 10, 85100 Potenza, Italy; 2 Dipartimento di Scienze, Università della Basilicata, via dell’Ateneo Lucano 10, 85100 Potenza, Italy; 3 Dipartimento delle Culture Europee e del Mediterraneo, Università della Basilicata, via Lanera 20, 75100 Matera, Italy

**Keywords:** *Trichoderma afroharzianum* T-22, Simulated wastewater effluent, Salinity stress, Irrigation, Aphids, Antioxidant, Plant growth

## Abstract

The scarcity of freshwater for agriculture in many regions has led to the application of sewage and saline water for irrigation. Irrigation with non-conventional water sources could become a non-harmful process for plant cultivation, and the effects of their use on crops should be monitored in order to develop optimal management strategies. One possibility to overcome potential barriers is to use biostimulants such as *Trichoderma* spp. fungi. Tomato is a crop of great economic importance in the world. This study investigated the joint effects of *Trichoderma afroharzianum* T-22 on tomato plants irrigated with simulated unconventional waters. The experiment consisted of a control and three water treatments. In the control, the plants were watered with distilled water. The three water treatments were obtained by using an irrigation water added with nitrogen, a wastewater effluent, and a mixed groundwater-wastewater effluents. Potted tomato plants (variety Bobcat) were grown in a controlled growth chamber. Antioxidant activity, susceptibility to the aphids *Macrosiphum euphorbiae*, and tomato plant growth parameters were estimated. *Trichoderma afroharzianum* T-22 had a positive effect on plant growth and antioxidant defenses when plants were irrigated with distilled water. Instead, no significant morphological effects induced by *T. afroharzianum* T-22 on plants were observed when unconventional water was used for irrigation. However, inoculation with *T. afroharzianum* T-22 activated a stress response that made the colonized plants more susceptible to aphid development and increased their fecundity and longevity. Thanks to this study, it may be possible for the first time to open a new discussion on the practical possibility of using reclaimed wastewater for crop irrigation with the addition of a growth-promoting fungal symbiont.

**Supplementary Information:**

The online version contains supplementary material available at 10.1007/s11356-024-32407-w.

## Introduction

One of the most urgent recommendations from the European Community and the Organisation for Economic Co-operation and Development (OECD) is to increase efforts to reduce freshwater consumption and preserve this natural resource, which is becoming increasingly scarce (Directive EU 2020; OECD 2023a). Avoiding environmental contamination and withdrawing freshwater for irrigation has become imperative. An increasingly widespread initiative is the recycling of treated wastewater. To this end, it is necessary to improve wastewater treatment technologies by reducing the content of organic and inorganic contaminants. It may therefore be necessary to enhance wastewater treatment plants (WWTPs) by implementing tertiary stages capable of removing even recalcitrant pollutants (OECD 2023a). It may be useful to plan irrigation trials on widely cultivated crops to obtain information on the practical feasibility of reusing wastewater and to test their resistance to pests. In the case of excessive salt accumulation in the soil, growth-promoting bacterial or fungal inoculants can be used to mitigate the negative effects of this stress (OECD 2023b).

One of the most worrying limitations of wastewater recycling in agriculture may be its excessive salinity, which is often not reduced by the primary and secondary treatment typically applied in WWTPs (Jaramillo and Restrepo 2017). It is documented that about 20% of all arable land is under severe salinity stress (Shahid et al. 2018). Salts accumulate in the soil when water used for irrigation is lost by high evaporation from the soil surface, as its removal by aerial parts of crops is insignificant (Qadir et al. 2001). Salt-affected soils are mostly found in arid and semi-arid regions, but their occurrence is likely to increase as a result of climate change. Salinity has several detrimental effects on plants exposed to this stress. Two phases of plant responses to salinity stress can be distinguished. The first one is a rapid ion-independent growth reduction that causes stomatal closure and inhibition of shoot cell expansion; the second one takes place over days or weeks and is related to cytotoxic ion levels, causing premature senescence and ultimately cell death (Munns et al. 2002; Roy et al. 2014; Isayenkov and Maathuis 2019). Plant growth, plant weight, number of leaves, photosynthetic pigments, and protein content are negatively affected by salinity (Kumar et al. 2017). In plants, high salt levels are associated with oxidative stress due to the generation of reactive oxygen species (ROS), leading to protein, lipid, DNA, chlorophyll, and cell damage (Bai et al. 2018; Isayenkov and Maathuis 2019; Mohammadi et al. 2021). Therefore, detoxification of ROS is a potential strategy for salinity tolerance. Tomato plants produce enzymatic and non-enzymatic compounds to prevent harmful effects of ROS, and the production of antioxidants can vary with salt concentration (Roșca et al. 2023). Under abiotic (drought, salinity, etc.) and biotic (pests and diseases) stress, the accumulation of secondary metabolites such as phenolic compounds, flavonoids, and proline is essential for plant survival (Ghodoum Parizipour et al. 2021).

Exposure to elevated salinity stress can shape the population dynamics of insect pests in an agroecosystem by altering plant growth and physiology (Roșca et al. 2023). Plants under drought or salinity stress increase their chemical defenses, reducing the nutritional quality for herbivorous insects, with positive, negative, or non-significant effects on pest populations, depending on the insect species and host plant (Huberty and Denno 2004; Rivelli et al. 2013; Dong et al. 2020; Ghodoum Parizipour et al. 2021; Shannag et al. 2021). For example, in the model system of tomato plants and the potato aphid *Macrosiphum euphorbiae* (Thomas), salinity stress negatively affected plant growth, aphid survival, and fecundity via cascading effects (Dong et al. 2020). The same reduction in survival and fecundity was observed in the cereal aphid *Rhopalosiphum padi* L. feeding on salt-stressed wheat plants, which may be related to an increase in the biosynthesis of phenolics and free proline in the plants (Ghodoum Parizipour et al. 2021).

The deleterious effects of salinity on tomato plants can be mitigated by strategies such as plant priming or genetic modification techniques. Avirulent opportunistic endophytic plant growth-promoting fungi of the genus *Trichoderma* promote resistance and mitigate the effects of biotic and abiotic stresses by producing phytohormones and antioxidants (Harman et al. 2004; Shoresh et al. 2010; Studholme et al. 2013). *Trichoderma* spp. inoculation improves plant resistance to moderate salinity stress in the medium term by promoting root development, increasing antioxidant activity, and enhancing photosynthetic capacity (Cheng et al. 2023). For example, maize (*Zea mays* L.) plants inoculated with *T. asperellum* increased their salt tolerance by reducing the symptoms of oxidative damage. The fungus improved the ionic balance, altered the expression of genes related to oxidative stress, increased the amount of non-enzymatic antioxidants, and thus reduced the accumulation of reactive oxygen species (Fu et al. 2017). In vitro and greenhouse experiments have shown that *T. asperellum* F-01763 increases the total proline content of tomatoes and this reduces salt stress (Kashyap et al. 2020). Plants colonized by *T. harzianum* changed their metabolic machinery, showing higher levels of compounds such as peroxidases and phenols, giving the plant a lasting resistance to stress (Harman et al. 2004). In maize, *Trichoderma* spp. have been shown to reduce the adverse effects on plant growth under moderate salinity stress conditions (Kumar et al. 2017). *Trichoderma virens* and *T. atroviride* promote plant growth in *Arabidopsis* under salinity stress through auxin signaling, inducing lateral roots and root hairs (Contreras-Cornejo et al. 2014).

Plants are the first level of the trophic chain that includes herbivorous insects and predators, and the effect of *Trichoderma* colonization on plant-insect interactions is controversial. Aphids of the species *M. euphorbiae* developed on tomato plants colonized by *T. longibrachiatum* MK1 showed an increase in their population growth due to the increased nutritional value of the plant. However, their predators and parasitoids are also more attracted to inoculated plants (Battaglia et al. 2013). On the other hand, long-term survival of *M. euphorbiae* was significantly reduced in tomato plants colonized by *T. atroviride* as a result of the upregulation of genes encoding for protective enzymes belonging to the oxidative defense compartment (Coppola et al. 2019a). Similar results were obtained by Coppola et al. (2019b) with *T. harzianum* T-22 (subsequently re-identified as *T. afroharzianum* T-22). To date, the combined effects of irrigation with unconventional water and *Trichoderma* inoculation on plant and aphid performance in tomato have not been documented.

In this study, we investigated the effects of irrigation with simulated unconventional waters on potted tomato plants and the potential use of the endophytic plant growth-promoting fungus *T. afroharzianum* strain T-22 to mitigate the adverse effects. The experiment consisted of a control and three water treatments. In the control, the plants were watered with distilled water. The three water treatments were obtained by using an irrigation water added with nitrogen, a wastewater effluent, and a mixed groundwater-wastewater effluent (Polo-López et al. 2012 with some modification). Municipal wastewater treatment plants typically treat wastewater with nitrate concentrations up to 200 mg/L NO_3_-N (Glass and Silverstein 1999). For example, soils in Mediterranean countries where wastewater is used for irrigation have a high N content (30 mg/L). In addition, Mediterranean soils that have been irrigated with wastewater for years have high conductivity (Ortega-Pozo et al. 2022). Nitrogen compounds that accumulate in the soil can lead to many potentially negative effects on plant growth as a result of overfertilization (Albornoz 2016). In the present experiment, we added large amounts of NO_3_ (about 470 mg/L) to the water treatments to get as close as possible to a real scenario where wastewater reuse is a common agricultural practice and, therefore, where there is a need to overcome the possible obstacle of salt accumulation in the soil.

Plant growth parameters and performance of *M. euphorbiae*, a common tomato insect pest causing direct damage (Walgenbach 1997) and virus transmission (Braithwaite and Blake 1961), were measured at different combinations of unconventional water irrigation and fungal inoculation. We also measured the response of the plant antioxidant defenses through the synthesis of specialized metabolites (polyphenols and flavonoids) and antioxidant activity (radical-scavenging activity and reducing power).

## Materials and methods

### Tomato plants and fungus inoculation

Seeds of cultivated tomato (*Solanum lycopersicum* L.) F1 hybrid line “Bobcat” were obtained from a commercial seed company (Syngenta, Italy). Seeds were kept for germination on wet cotton disks in the dark in sterile Petri dishes at 21 ± 1 °C and after 1 week were planted in small pots (2.5-cm diameter and 5-cm height) in alveolate containers with sterile soil (composition: peat, coconut fiber, and perlite) under controlled conditions in a climatic growth chamber at 21 ± 1 °C, 65 ± 5% relative humidity, with a photoperiod of 16:8-h light/dark.

The tomato seedlings were grown for 1 month until the first two true leaves appeared. During this phase, the seedlings were watered three times a week with tap water, and after 2 weeks, the plants were supplied with 10 mL of 0.3% NPK (7.5-3-6 + Fe and microelements) nutrient solution PIANTE VERDI (Compo®, Ravenna, Italia). At the same time, half of the pots were inoculated with *Trichoderma afroharzianum* T-22 (“*Trichoderma afroharzianum* T-22 colonization” section).

After 1 month, tomato seedlings, uniform in size, were transplanted into bigger biodegradable pots (7.5-cm height × 9.5-cm top diameter × 7-cm bottom diameter) containing 145 g of synthetic soil. The artificial soil was a mixture of peat (40% w/w), perlite (40% w/w), sand (10% w/w), and clay (10% w/w). The pots used in this study are made of wood fiber, peat, and cellulose and increase water dispersion compared to plastic pots. The plants were grown in a climate chamber at 23 ± 2 °C, 50 ± 5% relative humidity for 36 days. The lights used for plant growth consisted of a 20-W, 130-lm/W white LED tubes (6500 K) coupled with a 36 W, 100-lm/W full-spectrum LED tubes. A schematic representation of the experimental design is shown in Figure S1.

### *Trichoderma afroharzianum* T-22 colonization

Tomato plants were inoculated with the fungus *Trichoderma afroharzianum* Rifai strain KRL-AG2 T-22 (formerly *Trichoderma harzianum* T-22; Chaverri et al. 2015), a commercial purified strain that disperses in water (Trianum-P, KOPPERT B.V., Berkel en Rodenrijs, The Netherlands). Before starting the experiments, the viability of the commercial stock formulation of *T. afroharzianum* T-22 was confirmed in the laboratory by counting the number of colony-forming units after 24 h of incubation at 25 °C in the dark, as previously described by Forlano et al. (2022).

Two independent fungal inoculations were carried out on tomato plants. The first inoculation was carried out immediately after transplanting the germinated seeds into the pot containers. The germinated seeds were watered using a fresh solution of 4 g of the commercial product dissolved in 1 L of distilled water (1 × 10^9^ colony forming units/g of viable *T. afroharzianum* T-22 spores), 10 mL of solution per seed. Control seeds were treated with only distilled water. The inoculations were repeated after 3 weeks when the seedlings were transplanted into larger pots with synthetic soil.

The second inoculation was done immediately after transplanting; the seedlings were watered with 2 g of commercial product dissolved in 1 L of distilled water, 75 mL solution/pot. The control plants were watered using the same amount of distilled water. Based on our previous experiments (Caccavo et al. 2022; Forlano et al. 2022), 100% of fungal colonization was obtained following this experimental procedure; however, to ensure the presence of the symbiotic fungus throughout the experiment, fungal colonization was also confirmed by strain isolation on potato dextrose agar (PDA) medium. At the end of the experiments, a fraction of the tomato roots was collected from a sub-sample of eight control and eight inoculated plants (two per treatment). The roots of each plant were carefully washed to remove soil, cut into 2 cm long, immersed in a 70% hydroalcoholic solution, then in a 1% sodium hypochlorite solution, and finally washed with sterile distilled water. The prepared root was placed on Petri plates with PDA medium supplemented with streptomycin sulfate (0.05%). The plates were incubated for 7 days at 25 ± 1 °C, and the presence of the fungus was determined.

### Experimental design

The experiment consisted of a control and three water treatments set up with different electrical conductivity (EC). In the control treatment (Co-Dw), the plants were watered with distilled water. The three experimental water treatments were obtained by simulating (1) a water with added nitrogen (*S*_1_; 1.2 dS/m); (2) a wastewater effluent with nitrogen (*S*_2_; 1.4 dS/m); and (3) a mixed groundwater-wastewater effluent with nitrogen (*S*_3_; 1.95 dS/m). The EC of irrigation water was measured with Basic30, Crison. The exact compositions and the chemical properties of the water treatments are shown in Table 1.

**Table 1:** Experimental codes and physicochemical properties of experimental water used in this work

Experimental codes			Ionic content (mg/L)							EC (dS/m)	pH
Control	*Trichoderma afroharzianum*	Treatment	Na^+^	Mg^2+^	Ca^2+^	SO_4_^2−^	K^+^	NH_4_^+^	NO_3_^−^		
Co-DW	Tr-DW	DW									
Co-*S*_1_	Tr-*S*_1_	*S*_1_						135	465	1.2	6.57
Co-*S*_2_	Tr-*S*_2_	*S*_2_	31.2	12.9	19.3	93.9	4.45	136.5	469.7	1.4	7.56
Co-*S*_3_	Tr-*S*_3_	*S*_3_	58.9	25.2	38.6	184.6	7.94	136.5	469.7	1.95	7.84

The exact compositions are as follow: *DW*: distilled water; *S*_*1*_: NH_4_NO_3_ (600 mg/L); *S*_*2*_: NH_4_NO_3_ (600 mg/L), NaHCO_3_ (96.1 mg/L), NaCl (12.6 mg/L), CaSO_4_·2H2O (60.3 mg/L), CH_4_N_2_O (6 mg/L), MgSO_4_ (60 mg/L), KCl (15 mg/L), K_2_HPO_4_ (0.4 mg/L), CaCl_2_·2H_2_O (4 mg/L), MgSO_4_·7H_2_O (3.7 mg/L), urea (6 mg/L); *S*_*3*_: NH_4_NO_3_ (600 mg/L), NaHCO_3_ (192.2 mg/L), NaCl (15.2 mg/L), CaSO_4_·2H_2_O (120.6 mg/L), CH_4_N_2_O (12 mg/L), MgSO_4_ (120 mg/L), KCl (30 mg/L), K_2_HPO_4_ (0.8 mg/L), CaCl_2_·2H_2_O (8 mg/L), MgSO_4_·7H_2_O (7.4 mg/L), urea (6 mg/L)

To approximate real conditions, irrigation with experimental water began 2 days after the seedlings were transplanted to larger pots. After transplanting, the plants inoculated with *T. afroharzianum* T-22 (coded as Tr) and the control plants (Co) were divided into four groups that were periodically watered with an equal amount of experimental DW, *S*_1_, *S*_2_, and *S*_3_ water. Eight experimental treatments were generated, coded as Co-DW, Co-*S*_1_, Co-*S*_2_, Co-*S*_3_, Tr-DW, Tr-*S*_1_, Tr-*S*_2_, and Tr-*S*_3_: the first part of the code refers to the fungal inoculation and the second part refers to the water treatment (Table 1).

At each watering, one plant was watered with 150 mL of the appropriate water solution. Watering requirements over time were set to maintain soil moisture between 30 and 90% of water-holding capacity in all pots. After transplanting, nine irrigation treatments were applied over 36 days, with each plant receiving a total of 1350 mL of experimental water, generating three independent experimental lines.

The experiments were carried out in three separate periods, 14 days apart. This procedure resulted in three independent replicates of 10 plants each, giving a total of 30 plants for each of the eight experimental groups. For the experimental measurements, a sub-sample of 23 plants was used for plant growth over time (20 for Co-DW and Tr-DW treatments), 10 plants were used for aphid experiments, and 17 plants were used for plant morphological measurements (20 for Co-DW and Tr-DW treatments).

### Plant growth-related parameters

The growth of 21 seedlings per experimental treatment was monitored by measuring the height of the plant (*H*; cm) once a week at 7, 13, 19, and 26 days after transplanting (DAT). At 36 DAT, prior to cutting the plants, in addition to the height of the plant, the leaf chlorophyll content of a sub-sample of 17 tomato plants from each group was estimated by using a handheld device, the Soil Plant Analysis Development - SPAD 502 meter (Konica-Minolta Corporation, Ltd., Osaka, Japan). For each plant, average SPAD index value was calculated from three readings taken from the tip to the base of all fully expanded, non-necrotic leaves. Plants were then cut, separated into stem and leaves, and weighed to determine the total fresh weight (FW; g). Leaves were scanned to measure the leaf area (LA; cm^2^) using a LI-COR leaf area meter (Model 3100, Inc., Lincoln, NE, USA). Finally, the plants were dried in a ventilated oven at 70 °C until they reached a constant weight.

### Determination of antioxidant activity

Dried leaf tissues from plants of each replicate were pooled and used for three independent extracts per experimental group. Extraction was performed by using ultrasound-assisted extraction (Branson 1800 sonicator, frequency of 40 Hz and amplitude of 100%). Aliquots of dried leaves (1.5 g) were extracted using 50% ethanol/water as solvent in a ratio of 1:10 plant matrix:solvent for 30 min in the dark (with temperature control). After extraction, centrifugation was performed at 4500 rpm for 10 min at 21 °C and the extract was filtered through 0.2-μm filters and dried using a rotary evaporator. The antioxidant activity was evaluated by three different chemical-based assays (Karadag et al. 2009): the radical scavenging activity by 2,2-diphenyl-1-picrylhydrazyl (DPPH) and by 2,2′-azino-bis(3-ethylbenzothiazoline-6-sulfonic acid) (ABTS) and the reducing power by Ferric-Reducing Antioxidant Power (FRAP).

DPPH scavenging activity was determined as described in Mezrag et al. (2017). Different concentration of extracts or negative control (50 μL) was mixed by methanol DPPH solution (200 μL, 0.1 mM) and left at the dark for 30 min. Trolox (4.00–80.00 μg/mL) was used as reference standard.

ABTS scavenging activity was measured as described by Faraone et al. (2018). The generation of ABTS radical occurs after 16 h of incubation of ABTS salt and potassium persulfate at room temperature. Extracts (different concentrations) or negative control (15 μL) were incubated with 235 μL of ABTS radical and after 2 h of incubation, the absorbance was read at 734 nm. Trolox (5.00–100.00 μg/mL) was used as reference standard.

FRAP reagent was prepared by mixing 300 mM sodium acetate buffer at pH 3.60, 20 mM FeCl_3_·6H_2_O in distilled water, and 10 mM TPTZ in 40 mM HCl (10:1:1 ratio). Extracts or negative control (25 μL) were mixed with fresh FRAP reagent (225 μL) and after 40 min of incubation at 37 °C the absorbance was measured at 593 nm. Trolox (2.50–250.00 μg/mL) was used as reference antioxidant standard.

DPPH, ABTS, and FRAP values were expressed as milligrams of Trolox equivalents per gram of dried extract (mg TE/g) (Mezrag et al. 2017).

In addition, the total phenolic content (TPC) was determined by using the Folin–Ciocalteau (FC) assay described by Lamorte et al. (2018). An aliquot of extract (75 μL) at different concentrations was mixed with 500 μL of FC reagent and 500 μL of Na_2_CO_3_ (10×) and distilled water was added to reach a final volume of 1.5 mL. The reaction mixture was incubated for 1 h in the dark and the absorbance at 723 nm was measured using a UV-Vis spectrophotometer. Gallic acid (2.50–250.00 μg/mL in methanol) was used as standard compound and results were expressed as milligrams of gallic acid equivalents per gram of dried powdered sample (mg GAE/g).

The total flavonoid content (TFC) was determined by the colorimetric method (Lamorte et al. 2018). An aliquot of 125 μL extract was mixed with 125 μL of AlCl_3_. The UV-Visible spectrophotometer was used to measure the absorbance at 415 nm to determine the TFC concentration. Quercetin was used as standard compound (2.00–200.00 μg/mL in methanol), and results were expressed as milligrams of quercetin equivalents per gram of dried powdered sample (mg QE/g).

All spectrophotometric measurements were performed using a UV-Vis spectrophotometer (SPECTROstar^Nano^ BMG Labtech, Ortenberg, Germany).

### Aphid fecundity and survival

A colony of the aphid *Macrosiphum euphorbiae* was reared in the laboratory on tomato plants in a climatic box at a constant temperature of 22 ± 1 °C, 65% ± 10% relative humidity, and a photoperiod of 17:7, light and dark, respectively. For each replicate, approximately 200 virginoparae females were isolated from the mass-rearing colony and placed on two fresh potted tomato plants in a plastic box (30 × 30 × 30 cm) for 24 h in the chamber under the above-mentioned climatic conditions. Adult females were then removed and discarded. The new-born nymphs were maintained as a synchronous colony on the tomato plants for 10 days until they became adults.

Three adult females of the same age were then placed on 10 experimental 36 DAT tomato plants (3 plants for the first and third replicates, 4 for the second replicate) at 23 ± 2 °C. The aphids were left to feed on the experimental plants for 24 h, after which a fully expanded tomato leaf was covered by a clip cage (2-cm diameter), and two of the three parthenogenetic apterous adults were placed on the caged leaf for the fecundity experiments. Adult aphids were left in the clip cage on the plants for 24 h, after which the cage was opened, and the newly born aphid nymphs were counted. Aphid fecundity was then estimated as the mean number of nymphs per aphid.

For the survival experiments, two newly born aphid nymphs of each plant were placed on a leaf in a different position and covered with a clip cage. Aphid mortality was assessed at 1, 4, 6, 9, 11, and 13 days.

### Statistical analysis

Plant height over time (from 7 to 36 DAT) of each experimental group was fitted to a second-degree polynomial and analyzed by ANCOVA to test for differences among treatments.

The measurements of LA, FW, SPAD, antioxidants (ABTS, DPPH, FRAP, TFC, and TPC), and aphid fecundity were independently analyzed using linear mixed-effect models (LMMs) fitted with REML (restricted maximum likelihood), with “inoculation” (two levels: control and *T. afroharzianum* T-22), “water treatment” (4 levels, DW, *S*_1_, *S*_2_, and *S*_3_) as a fixed factor, and “replicate nested in inoculation and water treatment” as the random effect. The *P*-values for differences between inoculation and water treatment and their interaction were obtained by ANOVA (type II tests). The assumptions of homoskedasticity and normality were checked and met for this dataset (Shapiro-Wilk tests).

For all statistical models, the two main effects, the interaction term, and the random factor were fitted for each dataset. A backward procedure was used to sequentially remove non-significant effects, allowing the identification of the most parsimonious model.

A Cox proportional hazard regression model was used for the aphid longevity analysis to test for differences among treatments (likelihood ratio test).

The data were analyzed using the statistical software R (R Core Team 2022) with the packages lme4 (Bates et al. 2015) and lmerTest (Kuznetsova et al. 2017).

## Results and discussion

### *Trichoderma afroharzianum* T-22 root colonization

The success of the fungal colonization 36 days after transplanting was verified in a sub-sample of tomato plants. *Trichoderma afroharzianum* T-22 was isolated on PDA from all treated tomato plants, whereas the fungus was not observed in the control Petri plates (Fig. S2).

### Plant measurements

**Fig. 1:**
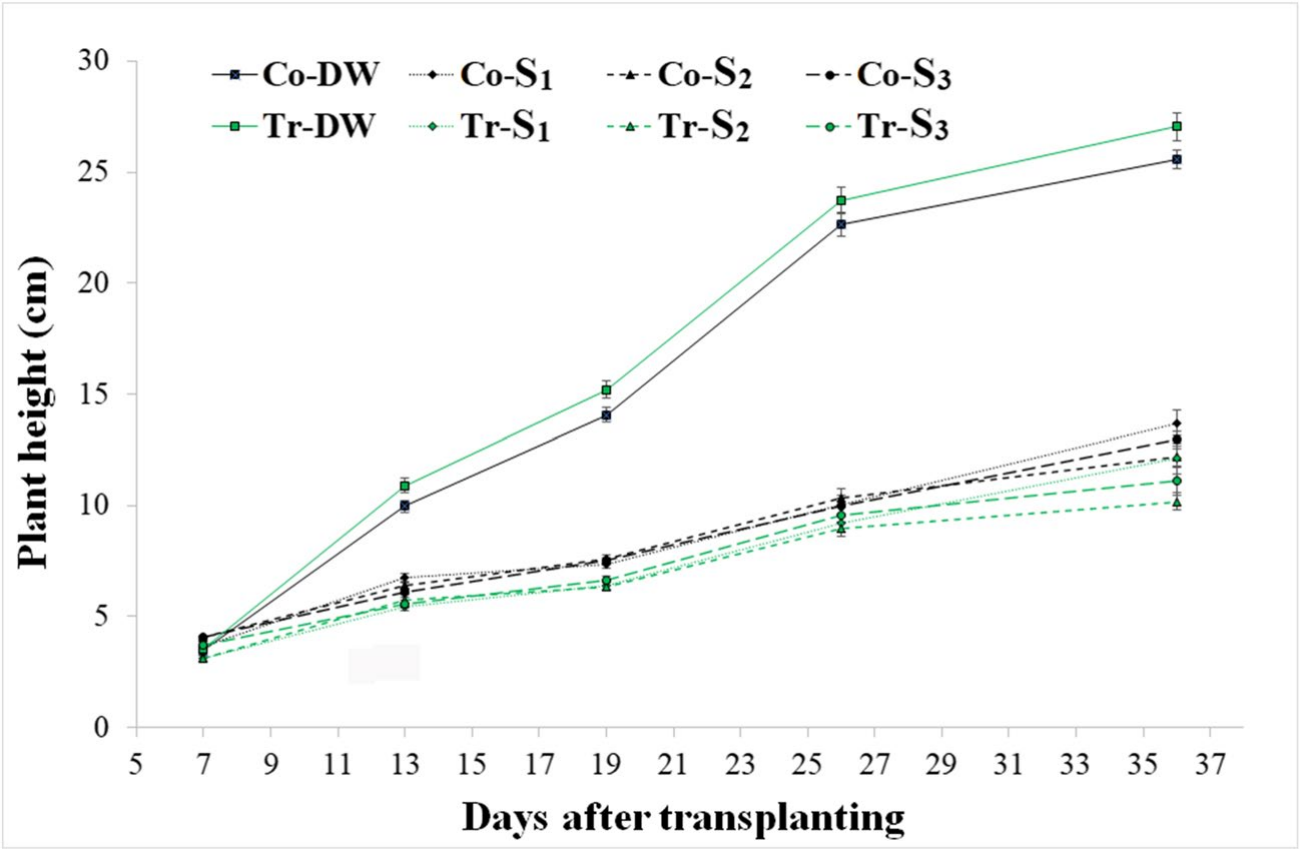
Plant height over time of the control and colonized tomato plants watered with four different water treatments. Co: control plants; Tr: plants colonized by *T. afroharzianum* T-22; DW: plants watered with distilled water; *S*_1_, water with added nitrogen (1.2 dS/m); *S*_2_, simulated wastewater effluent (1.4 dS/m); *S*_3_, simulated mixed groundwater-wastewater effluent (1.95 dS/m)

Figure 1 shows the plant height from day 6 to 36 after transplanting. The best fit for the plant height over time was given by two-degree polynomials (*R*^2^ > 0.97 in all the cases). The ANCOVA performed on this data showed significant differences in growth rates among treatments (*F*_14,813_ = 124, *P* < 0.001). Plants irrigated with distilled water grow faster than plants irrigated with unconventional waters as a result of salt accumulation. Also, in the *S*_1_ treatment, the higher nitrogen concentration induced overfertilization, which in turn reduced plant growth. Plants colonized by *T. afroharzianum* T-22 and irrigated with DW grow faster than their respective controls, whereas when *S*_1_, *S*_2_, and *S*_3_ water treatments were applied, control plants seem to perform better than inoculated plants.

**Fig. 2:**
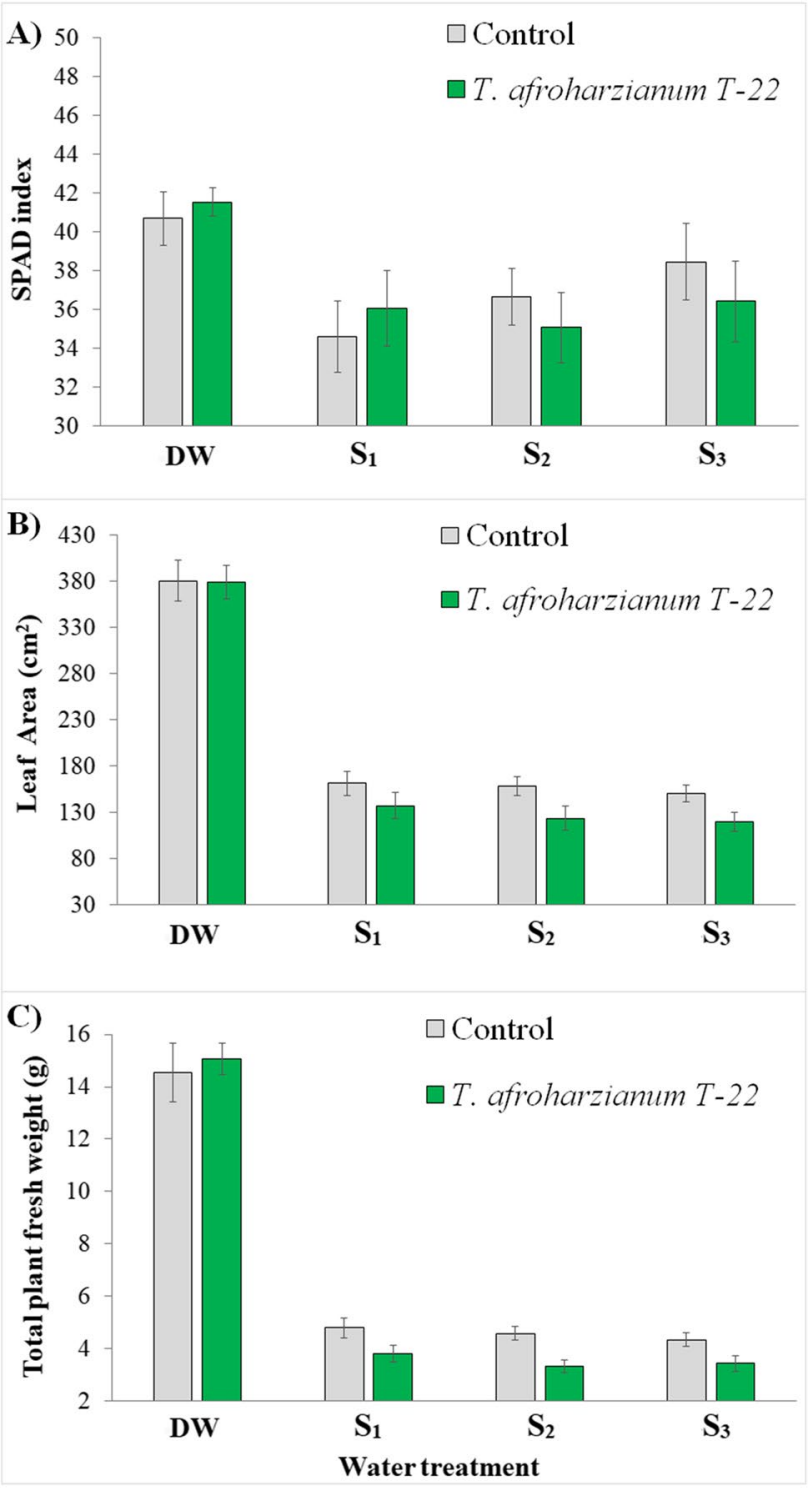
Mean values (±standard errors) of **A** of SPAD index, **B** leaf area, and **C** total plant fresh weight in relation to fungal inoculation and water treatment measured 36 days after transplanting. DW: plants watered with distilled water; *S*_1_, water with added nitrogen (1.2 dS/m); *S*_2_, simulated wastewater effluents (1.4 dS/m); *S*_3_, simulated mixed groundwater-wastewater effluents (1.95 dS/m)

Thirty-six days after transplanting, *T. afroharzianum* T-22 or irrigation with different simulated unconventional water had no effect on the SPAD index (Fig. 2(A)). Leaf area (LA) and total plant fresh weight (FW) were instead influenced by *T. afroharzianum* T-22 and irrigation water treatments, but the interaction between factors was never found to be significant (Table 2; Fig. 2(B) and (C)). Control plants exposed to different water treatments had higher LA values and were bigger than plants colonized by *T. afroharzianum* T-22. No significant effect of fungal colonization on LA was observed in DW plants.

**Table 2:** Results of GLMs testing the effects of “inoculation” and “water treatment” on leaf area (LA) and total plant fresh weight (FW)

*Effect*	LA			FW	
	d.f.	MS	*F*	MS	*F*
Inoculation	1	18,46	6.56*	17.33	5.6*
Water treatment	3	368,47	131***	748.6	243***
Residuals	120	3.14		2.18	

The random effect of replicates was never found to be significant

*d.f.*, degrees of freedom; *MS*, mean square; *F*, variance ratio

*P < 0.05; ***P < 0.001

Our results showed that *T. afroharzianum* T-22 begins to be effective in promoting tomato growth in plants watered with distilled water but failed to promote plant growth when the simulated unconventional waters (*S*_1_, *S*_2_, and *S*_3_) were used. The same trend was observed for other plant measures recorded in this experiment, such as LA and FW. Salinity is known to reduce plant shoot in several ways: by reducing photosynthesis, SPAD index, sugars in expanding tissues, by down-regulating long-distance signaling, and by changing the concentrations of specific ions (Rawat et al. 2011; Mohammadi et al. 2021; Roșca et al. 2023). It has been documented that *Trichoderma* inoculation improves plant resistance to moderate salinity in the medium term (Fu et al. 2017; Kashyap et al. 2020). However, relevant studies on this topic are limited and the results are often influenced by different factors such as the type and duration of salinity stress, the plant species, and the *Trichoderma* species (Cheng et al. 2023). Like other ascomycete fungi, *T. afroharzianum* T-22 could be considered to be an opportunistic plant symbiont. The plant responds to the *Trichoderma* colonization by activating local and systemic defense mechanisms with the aim of limiting the penetration of the fungus into the root. Once an equilibrium has been achieved, the interactions between the two organisms could be considered mutualistic, as *Trichoderma* provides benefits to its host plants (increasing nutrient availability and improving stress tolerance), while the fungus obtains organic compounds (Shoresh et al. 2010; Macías-Rodríguez et al. 2020). As environmental stress increases, the plant is forced to use all resource-consuming defense mechanisms to survive, leading to the phenomenon of the “growth-defense trade-off” (He et al. 2022). It is possible that the prolonged moderate-severe salinity stresses simulated under our experimental conditions threatened the mutualistic interactions between *T. afroharzianum* T-22 and tomato plants. We hypothesize that *Trichoderma* colonization may have been perceived by the plant as an additional source of stress, leading to a breakdown of mutualism and a transition to parasitism (Drew et al. 2021). This hypothesis is consistent with the study of Tan et al. (2022), who suggested that in the *Nicotiana benthamiana*-T. *harzianum* TRA1-16 model system, under low light intensity, *Trichoderma* mutualism turned into parasitism, reducing plant growth.

### Antioxidant defense and activity

**Fig. 3:**
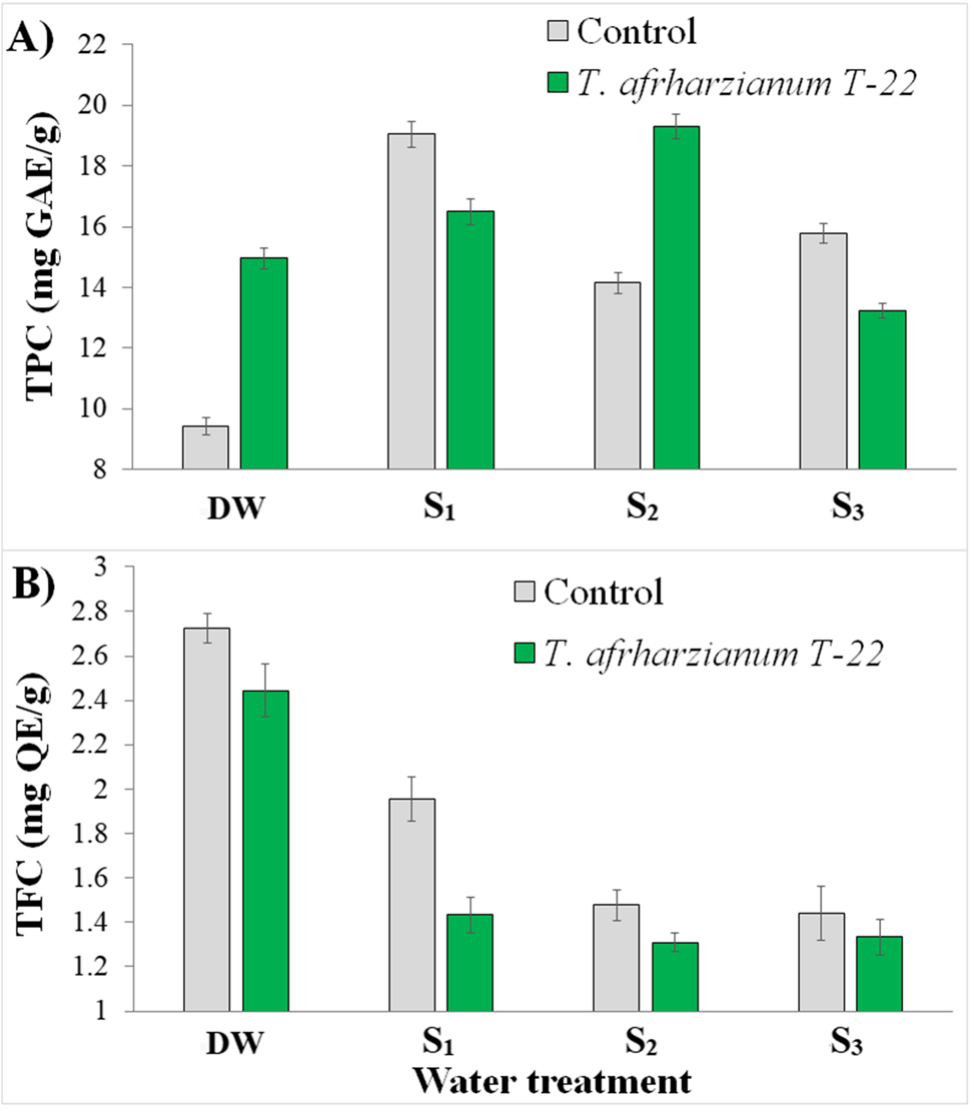
Mean values (±standard errors) of **A** total polyphenol (TPC) and **B** flavonoid content (TFC) measured in tomato plants in relation to fungal inoculation and water treatment 36 days after transplantation. DW: plants watered with distilled water; *S*_1_, water with added nitrogen (1.2 dS/m); *S*_2_, simulated wastewater effluents (1.4 dS/m); *S*_3_, simulated mixed groundwater-wastewater effluents (1.95 dS/m)

Significant effects of water treatment and *T. afroharzianum* T-22 on total phenolic content (TPC) were found in tomato plants (Fig. 3(A)). More interestingly, there was a significant interaction between the two factors (Table 3). Compared to the respective controls, *T. afroharzianum* T-22 induced an increase in TPC in the plants watered with distilled water and in those subjected to the *S*_2_ treatment (Tr-DW > Co-DW and Tr-*S*_2_ > Co-*S*_2_). An opposite trend was observed for the *S*_1_ and *S*_3_ treatments. TPC was found to be twice as high in Co-*S*_1_ than in Co-DW, but higher levels of salinity (*S*_2_ and *S*_3_) induced a gradual reduction in polyphenols (Fig. 3(A)).

**Table 3:** Results of LLMs testing the effects of “inoculation” and “water treatment” and their interaction on the ABTS, DPPH, FRAP, and TPC

*Effect*	ABTS			DPPH			FRAP			TPC			TFC		
	d.f.	MS	*F*	d.f.	MS	*F*	d.f.	MS	*F*	d.f.	MS	*F*	d.f.	MS	*F*
Inoculation	1	188.4	7.2**	1	15.79	5.02*	1	18.9	8.66**	1	45.5	21.6***	1	1.18	16.8***
Water treatment	3	651.3	24.9***	3	560.3	178.2***	3	340.5	156***	3	179.3	85.1***	3	5.38	76.9***
Interaction	3	569.3	21.8***	3	35	11.2***	3	32.2	14.8***	3	164.6	78.2***			
Residuals	190	26.11		164	3.14		200	2.18		123	2.1		62	0.0698	

The random effect of replicates was never found to be significant

*d.f.*, degrees of freedom; *MS*, mean square; *F*, variance ratio

*P < 0.05; **P < 0.01; ***P < 0.001

Total flavonoid content (TFC) was also influenced by water treatment and *T. afroharzianum* T-22 (Fig. 3(B); Table 3). Within a treatment, the control plants had higher TFC values than the inoculated plants. TFC decreased in plants irrigated with unconventional wastewater.

**Fig. 4:**
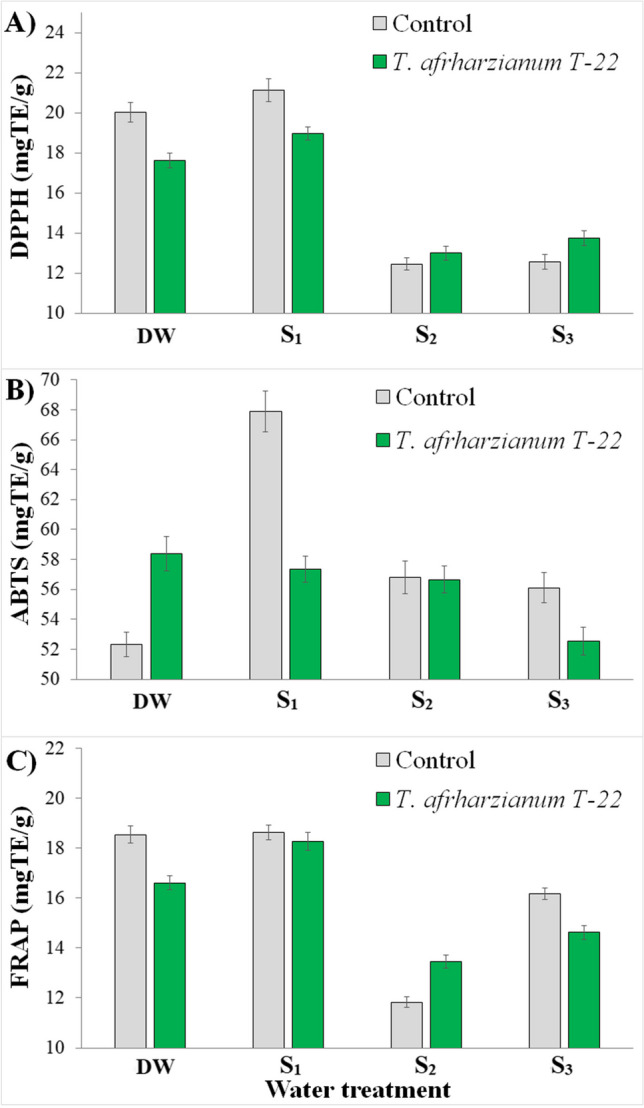
Mean values (±standard errors) of antioxidant activity measured in tomato plants in relation to fungal inoculation and water treatment 36 days after transplantation. **A** DPPH values; **B** ABTS values; and **C** FRAP values. DW: plants watered with distilled water; *S*_1_, water with added nitrogen (1.2 dS/m); *S*_2_, simulated wastewater effluents (1.4 dS/m); *S*_3_, simulated mixed groundwater-wastewater effluents (1.95 dS/m)

In terms of antioxidant activity (DPPH, ABTS, and FRAP), there were significant effects of water treatment and of *T. afroharzianum* T-22, as well as a significant interaction between the two factors (Fig. 4; Table 3). Regardless of the inoculum, DPPH values were similar between DW and *S*_1_ and between *S*_2_ and *S*_3_, with the former being higher than the latter. Control plants showed higher levels of DPPH than inoculated plants in the DW and *S*_1_ treatments, while the opposite trend was observed in the *S*_2_ and *S*_3_ treatments. Co-*S*_1_ plants had the highest value of ABTS, while Co-Dw and Tr-*S*_3_ had the lower value. ABTS values of the remaining groups are very similar. FRAP values were similar between DW and *S*_1_ and between *S*_2_ and *S*_3_, with the former being higher than the latter. Similarly, FRAP values recorded in control plants were higher than in inoculated plants in the DW and *S*_3_ treatments, lower in *S*_2_, and similar between each other in the *S*_1_ treatment.

Different scenario was observed using distilled water (DW) for irrigation; in fact, the total phenolic content and ABTS values were significantly increased after the fungus colonization. These results confirm that *T. afroharzianum* T-22 was conducive to enhancing the antioxidant activities in potted tomato plants (Harman et al. 2004; Shoresh et al. 2010; Studholme et al. 2013; Cheng et al. 2023). However, the higher antioxidant activity was observed in the *S*_1_ treatment, with Co-*S*_1_ showing higher values of antioxidant defense and activity (except for FRAP) than Tr-*S*_1_. Under our experimental conditions, simulated unconventional waters together with *Trichoderma* colonization could be considered as severe abiotic and biotic stresses for potted tomato and the ability of the plants to survive the stress is influenced by their antioxidant defense systems. Several studies have shown that salinity induces an oxidative condition due to the increased production of free radicals and the alteration in antioxidant systems (Zhang et al. 2019; Sofy et al. 2021). To reduce the damage caused by oxidative stress, plants have developed many antioxidant defense systems including enzymes, antioxidant ability (DPPH, ABTS, FRAP, etc.), and antioxidant molecules such as phenols and flavonoids (Azeem et al. 2023). Our results from the *S*_1_ treatment are consistent with these studies and confirm an increase in antioxidant activity (measured as total phenolic content, DPPH, ABTS, and FRAP). Plant height, leaf area, and FW revealed the existence of severe salinity stress in the potted tomato plants, but the ability to mitigate ROS in the *S*_2_ and *S*_3_ treatments was limited, probably as a consequence of the “growth-defense trade-off” phenomenon (He et al. 2022). However, under the *S*_1_, *S*_2_, and *S*_3_ water treatments, *T. afroharzianum* T-22 did not increase the plant antioxidant activity (except for TPC and FRAP), as previously reported (Adusumilli and Kolli 2022; Tyśkiewicz et al. 2022). These results seem to confirm the hypothesis that *Trichoderma* mutualism turned into parasitism under salinity stress.

### Aphid performance

**Fig. 5:**
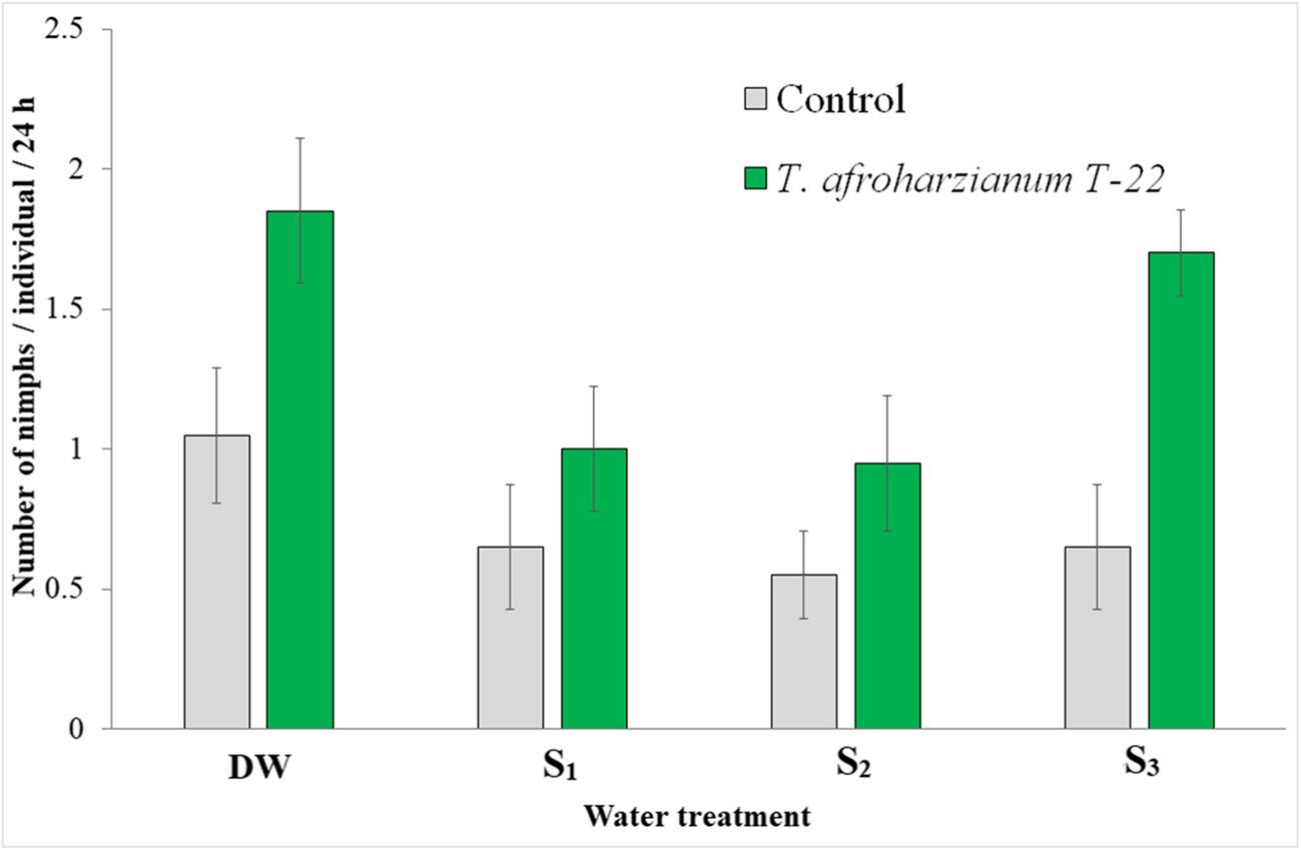
Aphid fecundity on tomato plants 36 days after transplantation. DW: plants watered with distilled water; *S*_1_, water with added nitrogen (1.2 dS/m); *S*_2_, simulated wastewater effluents (1.4 dS/m); *S*_3_, simulated mixed groundwater-wastewater effluents (1.95 dS/m)

Aphid fecundity was significantly higher on tomato plants colonized by *T. afroharzianum* T-22 (*F*_1,75_ = 17.6, *P* < 0.001) and decreased with irrigation with unconventional waters (*F*_3,75_ = 4.39, *P* < 0.01). However, the interaction between these two factors was never found to be significant (Fig. 5). More interestingly, aphid fecundity was statistically higher in the Tr-*S*_3_ group than in the control group.

**Fig. 6:**
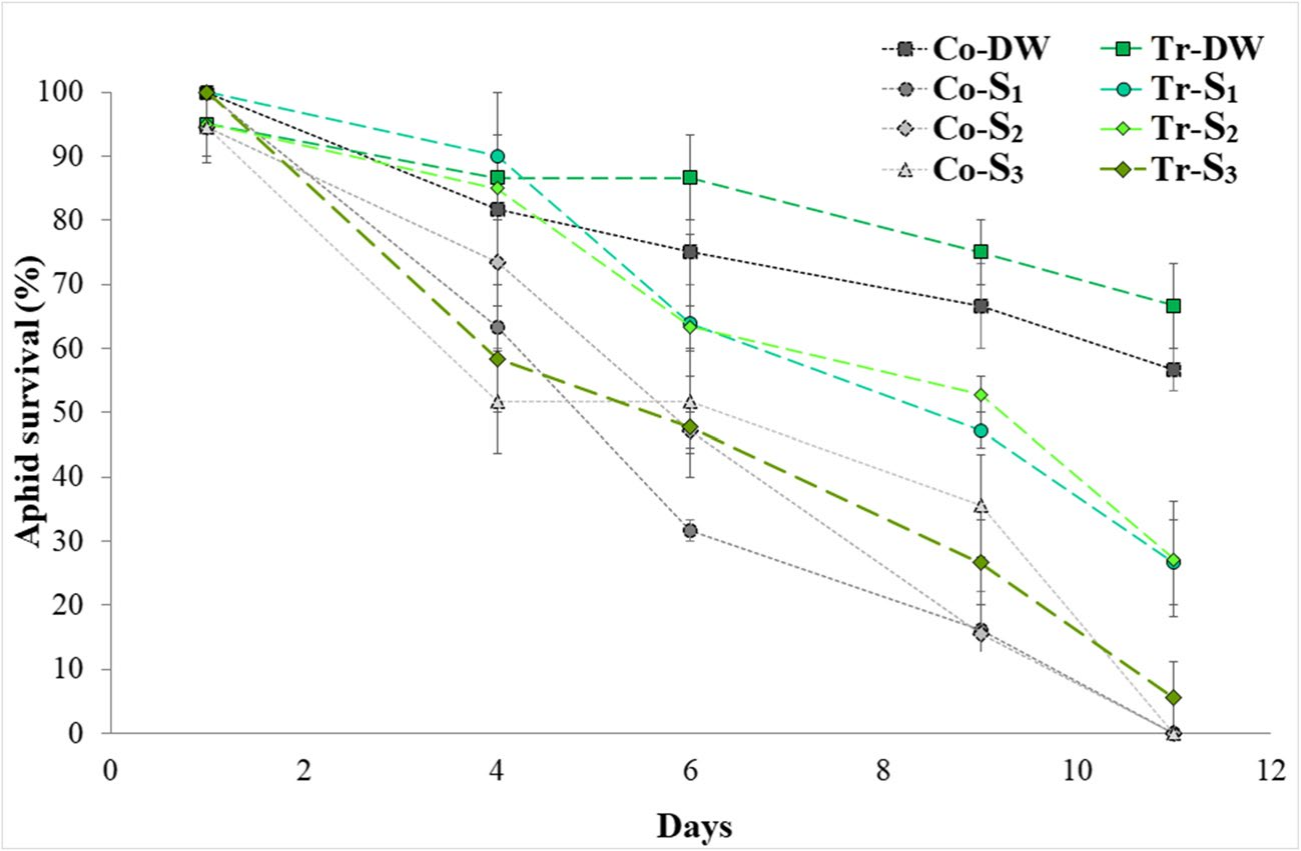
Survival over time of aphids reared on experimental tomato plants. Co: control plants; Tr: plants colonized by *T. afroharzianum* T-22; DW: plants watered with distilled water; *S*_1_, water with added nitrogen (1.2 dS/m); *S*_2_, simulated wastewater effluent (1.4 dS/m); *S*_3_, simulated mixed groundwater-wastewater effluent (1.95 dS/m)

The survival of aphids reared on experimental tomato plants over time is shown in Fig. 6. A Cox proportional hazard model identified significant treatment differences (likelihood ratio test: *χ*^2^ = 57.8, d.f. = 7, *P* < 0.001). Aphids that developed on plants irrigated with distilled water (Co-DW and Tr-DW) had a higher survival rate than the other experimental water treatments. The short-lived groups were the aphids that developed on plants of the Co-*S*_1_, Co-*S*_2_, Co-*S*_3_, and Tr-*S*_3_ groups. Aphids developed on Tr-*S*_1_ and Tr-*S*_2_ plants showed an intermediate longevity.

With the exception of the Tr-*S*_3_ treatment, aphid fecundity also decreased as a result of bottom-up effects of irrigation with unconventional waters. The unfavorable performance of the aphids may be related to the accumulation of toxic substances in the tomato leaves which reduce the feeding of the insects (Güntner et al. 1997; Han et al. 2016). In the *S*_1_ treatments, overfertilization stress resulting in smaller tomato plants with fewer and smaller leaves (Scholberg et al. 2000) may have caused the lower aphid performance. Our results support the “Plant Vigor Hypothesis” (Price 1991), which suggests that insect herbivores perform better on vigorous plants with higher nutritional value and lower chemical defenses.

It is documented that *Trichoderma* spp. induces a more efficient response of the plant against phytophagous insects (Hermosa et al. 2012; Di Lelio et al. 2021). Under field conditions, colonization of tomato plants by *T. harzianum* T-22 reduces the abundance of piercing-sucking insect pests and plant pathogens (Caccavo et al. 2022). In laboratory studies, *T. harzianum* T-22 has been shown to reduce the growth rate of the green stink bug *Nezara viridula* L. in tomato (Alınç et al. 2021). On the other hand, *M. euphorbiae* developed on potted tomato plants colonized by *T. longibrachiatum* MK1 showed an increase in their population growth due to the increased nutritional value of the plant (Battaglia et al. 2013). In our study, within each experimental treatment (including DW), *M. euphorbiae* showed higher fecundity and survival on the plants colonized by *T. afroharzianum* T-22. As suggested by Battaglia et al. (2013), the better performance of this insect pest on the plants colonized by *T. afroharzianum* T22 may be due to a higher nutritional value of the plants, at least for the DW treatment. *Trichoderma* spp. have been reported to enhance plant absorption of nutrients, especially in poor soils (Altomare and Tringovska 2011; Mastouri et al. 2012; Vinale et al. 2012). In accordance with several authors (Fu et al. 2017; Kumar and Ashraf 2017; Kashyap et al. 2020; Cheng et al. 2023), we can also conclude when the same unconventional water was used, inoculation with *T. afroharzianum* T-22 potentiate an antioxidant activity that makes the colonized plants more susceptible to aphid development, although no noticeable phenotypic effects on the plants were detected.

Although plant growth and antioxidant activity were reduced in the more stressed *S*_3_ treatment, surprisingly *T. afroharzianum* T-22 resulted in an increased aphid fecundity compared to *S*_1_ and *S*_2_ treatments, thus limiting the symptoms of abiotic stress on the insect. However, the same trend is not observed for aphid survival, with values very similar to those recorded for uninoculated plants exposed to salt stress. This result suggests that in the long term, colonization with *T. afroharzianum* T-22 is not very effective to allow significant recovery (and therefore detoxification) of the Tr-*S*_3_ plant, resulting in the observed detrimental effects on aphid survival.

## Conclusions

Irrigation with reclaimed wastewater and/or saline water has been reported to increase total soil nitrogen and, more generally, excessive soil salinity (Jaramillo and Restrepo 2017). The use of the fungus *Trichoderma* to mitigate the negative effects of stress and as a biocontrol agent for insect pests in agricultural systems has also become to be a real and practical possibility in agriculture (Poveda 2021; OECD 2023b). However, care must be taken to avoid excessive soil salinization, which may occur more frequently under climate change. Our results emphasize the need to improve wastewater treatment plants by implementing strategies capable of removing inorganic compounds. The use of growth-promoting fungal symbionts, such as *T. afroharzianum* T-22, to mitigate the negative effects of irrigation with unconventional water may be a successful strategy as it influences the production of antioxidants necessary to overcome mild stress. However, if the stress is more severe or prolonged, even colonization by *T. afroharzianum* T-22 will not be able to mitigate the deleterious effects on the plant. Tomato colonization by *T. afroharzianum* T-22 also appears to have a positive effect on aphid performance when irrigation with unconventional water occurs, confirming the increased vigor of the plants. In terms of pest control, the beneficial effect of *Trichoderma* colonization on tomato plants could almost be due to the strong attraction of aphid predators and parasitoids to the inoculated plants (Battaglia et al. 2013; Caccavo et al. 2022; Forlano et al. 2022).

### Supplementary information

Supplementary file 1 (DOCX 2557 kb)

## Data Availability

Data will be made available on request.
