# Supplementary material for: Wastewater irrigation and Trichoderma colonization in tomato plants: effects on plant traits, antioxidant activity, and performance of the insect pest Macrosiphum euphorbiae
Source: Environ Sci Pollut Res Int. 2024 Feb 14;31(12):18887–99. doi: 10.1007/s11356-024-32407-w (PMC10923738; doi:10.1007/s11356-024-32407-w)
Supplement: Supplementary file 1 — (DOCX 2557 kb) [file 11356_2024_32407_MOESM1_ESM.docx]

**Supplementary Information**


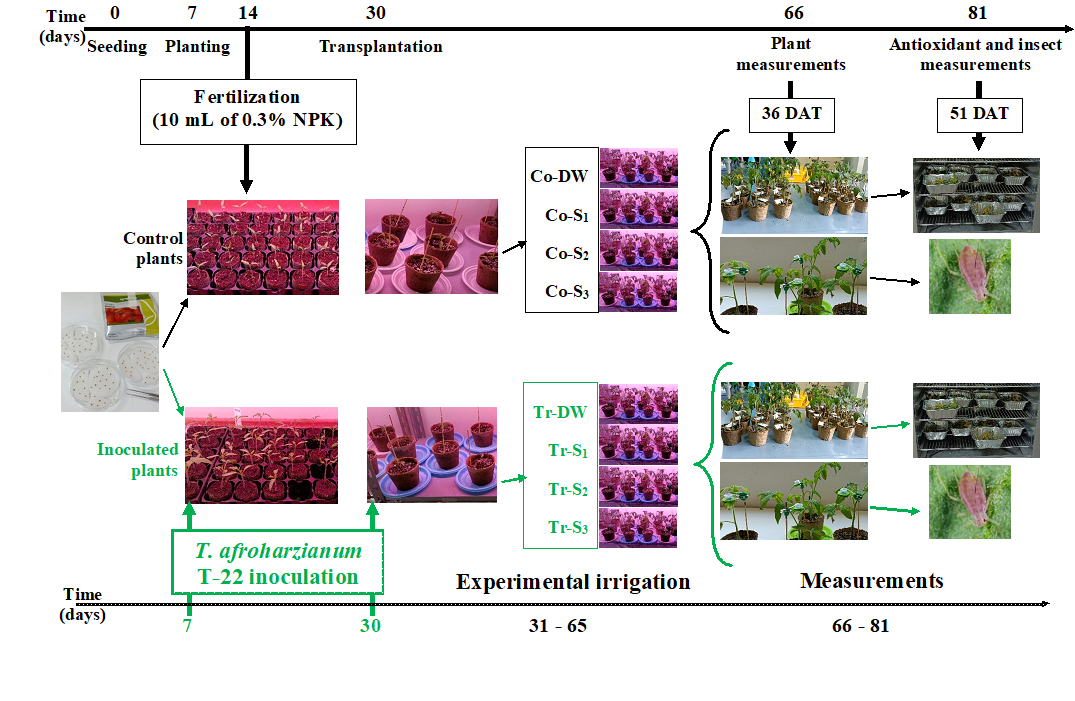
**Figure S1.**

**Figure S1. S**chematic representation of the experimental design. DAT: days after transplanting; Fertilization: the plants were supplied with 10 mL of 0.3% NPK (7.5-3-6 + Fe and microelements) nutrient solution PIANTE VERDI (Compo®, Ravenna, Italia).

**Figure S2.**


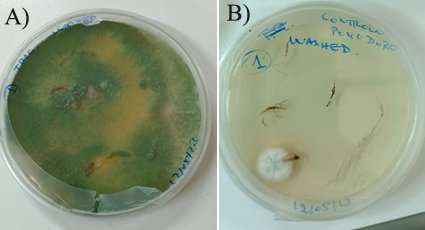


**Figure S2.** A) Photograph of a seven-day-old Petri plate with the roots of inoculated tomato plants showing the colony of *Trichoderma afroharzianum* T-22. B) Photograph of a seven-day-old Petri plate showing the roots of control plants.
